# Gender differences in health insurance coverage in China

**DOI:** 10.1186/s12939-021-01383-9

**Published:** 2021-02-01

**Authors:** Mei Zhou, Shaoyang Zhao, Zhi Zhao

**Affiliations:** 1 School of Public Administration, Southwestern University of Finance and Economics, Chengdu, Sichuan China; 2 School of Economics, Sichuan University, Chengdu, China

**Keywords:** Gender gap, Health insurance coverage, Health insurance reform, Education

## Abstract

**Background:**

China initiated a reform of the health insurance system in the late 1990s. The new insurance, Urban Employee Basic Medical Insurance (UEBMI), is employment-based, which makes it more difficult than it used to be for those unemployed or informal employed (most of whom are women) to be covered by health insurance.

**Methods:**

Based on three large sample of micro datasets, we first use statistical methods to identify gender differences in health insurance. Next, we construct a logistic regression model to capture the differences in insurance coverage across age groups using the parameter of interaction terms for gender and age groups.

**Results:**

Based on data from a demographic survey that covers a large sample, we find that in the below 50 (in 2005) or 60 (in 2015) years age group, the coverage gap of UEBMI between men and women was relatively smaller, while a larger disparity existed in the above 50 (in 2005) or 60 (in 2015) group. Moreover, gender differences in health insurance were more significant in the low-education group, while no gender differences were found in the high-education group.

**Conclusions:**

This paper explains the gender gap in health insurance and the reason for the wider gap among older people. Our study indicates that because the UEBMI in China mainly covers people with formal jobs, a lower labor participation rate (even much lower in formal jobs) of women has led to their greater difficulty in obtaining health insurance. Since the older women’s greater difficulty in obtaining jobs or susceptibility to lay-offs during the period of the UEBMI’s implementation, the possibility of being covered was even much lower. In fact, it was because of the combined effects of the UEBMI system and the labor market condition at that time that older women had a lower proportion of being covered under the UEBMI.

**Supplementary Information:**

The online version contains supplementary material available at 10.1186/s12939-021-01383-9.

## Background

The Chinese government has been trying to build a universal public social health insurance (SHI) system since the early 2000s, and has essentially achieved universal SHI coverage [1–4]. The ambitious SHI initiative consists of three key programs: the Urban Employee Basic Medical Insurance (UEBMI) for the urban employed, initiated in 1998; the New Cooperative Medical Scheme (NCMS) for rural residents, established in 2003; and the Urban Resident Basic Medical Insurance (URBMI), covering urban residents without formal employment (including children, the elderly, and other unemployed), launched in 2007. While enrollment under the UEBMI is compulsory for urban employees, the NCMS and the URBMI are voluntary insurance programs. By 2018, more than 97% of the entire Chinese population had SHI [5]. However, obvious inequities remain in the insurance system [6–9].

On the one hand, the UEBMI has the highest level of financing and benefits—significantly higher than those of the other two; On the other hand, there are inequities in the duration of health insurance coverage. It was not until the establishment of URBMI in 2007 that the accessibility of health insurance to urban residents was improved, especially for the unemployed women. In the last decade, health insurance coverage has resulted in large gender inequities, which may further affect the accessibility of health resources and health status. Therefore, this paper focuses on the gender difference in UEBMI coverage.

The roots of the inequity in China’s health insurance lies in the flawed system design of the UEBMI system reform. The UEBMI’s features of being employment-based and not including other family members stems from the 1994 reform of the health insurance system [10]. Before the reform, the healthcare insurance system in the era of the planned economy consisted of labor insurance and public health services. Public health services were mainly provided to part of the government staffs, whereas labor insurance covered general enterprise employees and their family members.

In 1994 China initiated the reform of the medical insurance system by implementing an SHI system compatible with the market economy—the UEBMI system. After several years of pilot work in Zhenjiang (Jiangsu province) and Jiujiang (Jiangxi province), this system was ultimately carried out nationwide in 1998. The UEBMI mainly covers the employed (excluding family members). Compared with the labor insurance system, the biggest change under the UEBMI is the requirement of being linked to one’s work unit, which makes it more difficult than it used to be for those unemployed or informal employed (most of whom are women) to be covered by medical insurance. Under the labor insurance system, a work unit’s medical insurance covered family members, although covering only half of their medical expenses. Meanwhile, under the new health insurance system, it is difficult for someone without a formal job to be covered by the UEBMI. Thus, compared with men, the situation has gotten tougher for women than ever before.

From the perspective of the UEBMI system reform in China, we investigate whether the basic health insurance coverage rate for women is lower than that for men and how this gender gap varies across the age groups. As for younger women and men, due to their higher employment rate, a higher proportion of them obtain the UEBMI. Meanwhile, for older women and men, health insurance is also available, provided they originally have a job. And it is mainly the group without a job that faces more difficulties in obtaining health insurance, within which women ought to account for the largest proportion. Women’s lower labor participation rate suggests that they have significantly lower access to health care than men do, especially the elderly. This is the gender difference in health insurance coverage that is examined in this study.

Based on the census data, this paper describes in detail the gap between women’s and men’s health insurance coverage. Our most important finding is that this gap manifests itself primarily in terms of age, where there is a large and persistent gender gap in the older age groups, and that the gender gap in health care coverage for younger people is not large due to the relatively high participation rate of Chinese women in the labor force. We describe the institutional context in which this disparity arises and analyze the potential impact of gender disparities in health insurance on health care utilization and health.

This study first confirms the existence of a gender gap in health insurance coverage based on a survey of a large population sample. Thereafter, we compare the changes in these gaps before and after the 1998 UEBMI reform using the China Health and Nutrition Survey (CHNS) data. Next, we discuss the possible reasons for inequities in the health insurance system and the potential impact on healthcare utilization and health status. Finally, we draw our conclusions and provide recommendations.

## Methodology

### Data sources

In this study, we used three datasets to validate gender differences in health insurance: one-fifth of the 2005 National Sample Survey of 1% of the population (randomly chosen; Sample A), the 2015 survey data of 1% of the population in Sichuan Province (Sample B), and the 1991–2011 survey data from the CHNS (Sample C).

We began by using Sample A. China conducts a population census every 10 years, and a large-scale population survey—the 1% of the population sample survey—is conducted between the two censuses. Sample A covers 2,585,481 individuals in 31 provinces, autonomous regions, and municipalities in Chinese mainland. Populations in both agricultural and non-agricultural areas are included in the survey. The survey covers respondents’ personal characteristics, including gender, age, and educational level, as well as other information such as the type of health insurance, health status, and so on. In the subsequent analysis, we further used Sample B. Samples B comprises 1% of the population of Sichuan Province only and comprises 1 million respondents.

To test our conjecture, we used Sample C, which covers eight waves of pre- and post-1998 reform data, enabling us to make a comparison of the changes in health insurance between men and women. The corresponding survey for Sample C covered nine provinces, which vary considerably in terms of geography, economic development, public resources, and health indicators. A stratified sampling scheme was used to stratify districts in the nine provinces by income, with four districts in each province randomly selected. The entire survey consists of about 4400 households, covering about 19,000 people. The survey participants are also tracked in follow-up surveys and some of them who could no longer be tracked due to migration and other reasons are replenished by adding new samples.

### Variables

The key dependent variable (*Insurance*) in our analysis relates to whether a person is covered by the UEBMI. *Insurance* is a binary dummy variable that is 1 if the individual is enrolled in UEBMI and 0 if they are enrolled in NCMS, URBMI, or do not have any insurance. It should be noted that this study focuses on whether they are covered by UEBMI and therefore classifies individuals who are NCMS, URBMI, or do not have any insurance as one category. In 2005, only two years after the establishment of NCMS, the participation rate was low and there were more uninsured individuals; by 2015, the percentage of uninsured was very low, and most people have been covered by UEBMI, NCMS or URBMI.

Age is an important grouping variable; thus, we removed samples under the age of 15 and separated the sample into age groups with 10-year intervals. We also conducted a heterogeneity analysis of the sample according to education level and compared the variability in insurance coverage based on education levels. In the regression analysis, we controlled for the sample’s personal characteristics such as gender, Hukou, marital status, and ethnicity. Hukou (a unique household registration system in China) is a dummy variable that refers to whether the survey respondent is registered in a rural or urban area. Classifications for marital status include married, unmarried, and widowed. Ethnicity is a dummy variable that denotes whether the sample is an ethnic minority or not.

### Estimation models

Since *Insurance* is a binary variable, we constructed a logistic regression model to investigate further the gender gap of health insurance coverage. The model is presented in Eq. (1) below:

1 $$ {y}_i={\beta}_1{age}_i\ast {female}_i+{\beta}_2{age}_i+{\beta}_3{female}_i+{X}_i\gamma +{u}_i $$

where the subscripts *i* denotes individual; *β* and *γ* are the estimated parameters, in which *β*_1_ is our key parameter that captures the differences in insurance coverage across age groups. The dependent variable, *Insurance*, denotes whether individual *i* is covered by UEBMI; *age* denotes the age group of individual *i*; *female* denotes the gender of individual *i;* and *X* denotes the other control variables, including Hukou, educational level, marital status, and regional fixed effects. The error term *u* denotes the random variability not explained by the model.

## Results

### Gender gap in health insurance coverage: based on survey data of 1% of the population in 2005 (sample a)

**Fig. 1:**
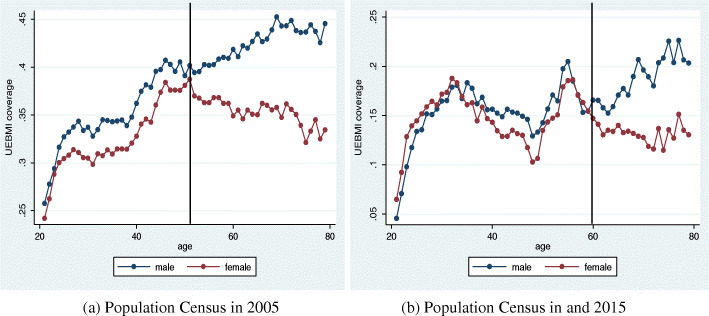
The Urban Employee Basic Medical Insurance (UEBMI) Coverage Rate: Based on Survey Data of 1% of the Population in 2005 and 2015

**Fig. 2:**
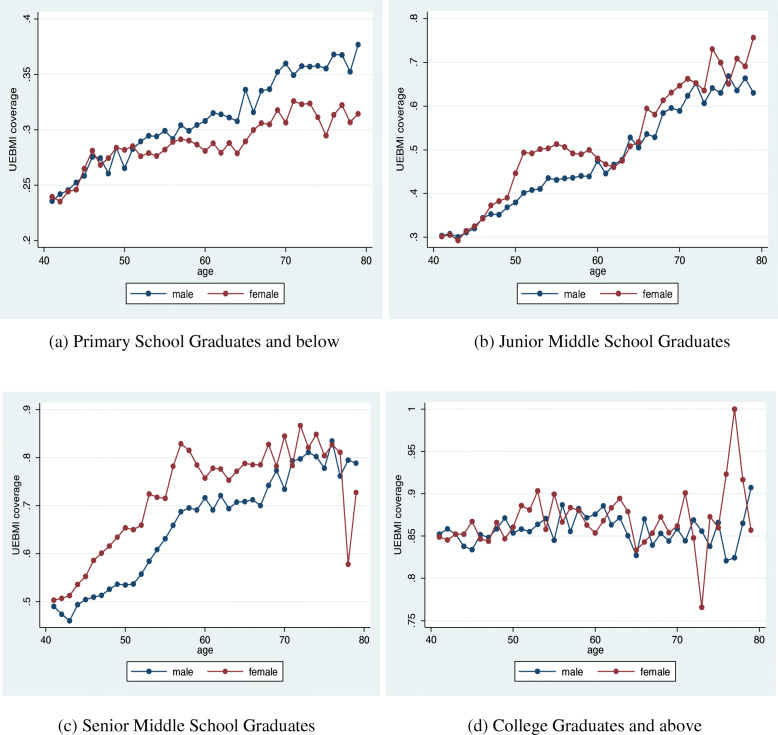
The Urban Employee Basic Medical Insurance (UEBMI) Coverage Rate in 2005 by Educational Levels

In the first stage of the analysis, we used Sample A. Figure 1 shows that for all age groups, the proportion of women covered by health insurance was lower than that of men. This was due to the higher labor participation rate of men. Meanwhile, by age group, the proportions of men and women with insurance coverage revealed different distribution trends. For men, the proportion of those covered by health insurance increased continually with age; while for women aged below 50 years, the proportion increased continually with age as well. However, this proportion kept declining continuously for the older women (aged above 50 years) and more obviously for those aged above 60 years. Considering the educational level (Fig. 2), the UEBMI coverage rates for men and women aged below 50 years were almost equivalent. However, for the groups aged over 50, for women, the proportion of those with insurance coverage was significantly lower than that for men only in the less educated group (primary school graduates and below), and this gap tended to widen with age. In general, this suggests that education level might have influenced the low rate of insurance coverage for the older people, while this effect of education did not exist for the younger people.

**Fig. 3:**
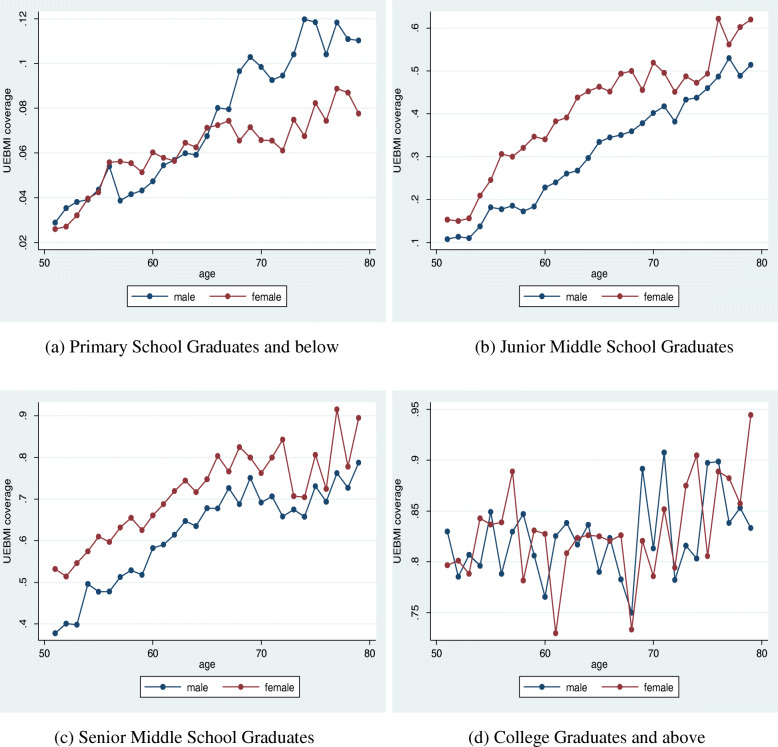
The Urban Employee Basic Medical Insurance (UEBMI) Coverage Rate in 2015 by Educational Levels

Next, in the regression estimation, we classified the population by age, with [50, 55) being the control group. Table 1 reports the result of the regression, in which columns 1 and 2 correspond to regression models with and without control variables (such as education), respectively. As shown in Table 1, when variables such as the educational level were not controlled for, the proportion of women being covered by UEBMI in all age groups was always lower than that of men (the coefficients of both female and female*age are significantly positive). This is consistent with the previous results as shown in Fig. 2. Moreover, when controlling for variables such as the educational level, the coefficient of women became significantly positive, indicating a higher proportion of women being covered by health insurance in the age group of [50, 55) as compared with men. However, since the other interaction term coefficients of gender and age are both significantly negative and the absolute values are greater than the coefficient of female, this indicates that generally, the women’s health insurance coverage rate was still significantly lower than that of men. By further observing the coefficients of the interaction term, we can clearly find their obvious changes below and above the age bracket of [50, 55). Below this age bracket, there is no clear trend in these coefficients of the interaction term, while above this age bracket they continue to increase in absolute value. This means that the health insurance coverage gap between women and men has been continuously widening for those above the age of 55.

**Table 1:** Results of Different Estimation Methods: Based on Population Census in 2005

Urban Employee Basic Medical Insurance (UEBMI) (Dependent Variable: Covered by UEBMI or Not)			
	(1)	(2)	(3)
Female	−0.0937*** (0.0098)	− 0.125*** (0.0106)	0.0548*** (0.0110)
Female *age [15,25)	0.0629*** (0.0135)	0.0690*** (0.0149)	−0.148*** (0.0161)
Female *age [25,35)	−0.0395*** (0.0119)	− 0.0300** (0.0131)	− 0.199*** (0.0138)
Female *age [35,45)	−0.0498*** (0.0115)	− 0.0484*** (0.0127)	− 0.119*** (0.0132)
Female *age [45,50)	− 0.00956 (0.0139)	− 0.00845 (0.0151)	− 0.0191 (0.0158)
Female *age [55,60)	− 0.0810*** (0.0151)	− 0.130*** (0.0163)	− 0.121*** (0.0169)
Female *age [60,65)	− 0.196*** (0.0166)	− 0.269*** (0.0179)	− 0.250*** (0.0186)
Female *age [65,70)	− 0.239*** (0.0175)	− 0.320*** (0.0188)	− 0.249*** (0.0197)
Female *age [70,75)	− 0.288*** (0.0189)	− 0.384*** (0.0203)	− 0.277*** (0.0211)
Female *age [75,80)	− 0.358*** (0.0227)	− 0.472*** (0.0241)	−0.344*** (0.0253)
Female *age [80 +1, 00)	−0.426*** (0.0253)	−0.531*** (0.0270)	− 0.426*** (0.0284)
X	No	No	YES
City	No	YES	YES
Observations	1,904,763	1,904,763	1,903,627

Notes: 1) The control age group is [50, 55)

2) The control variable X includes registered permanent residence, marital status, educational level, nationality, and so on. City represents fixed effects after controlling for cities

3) ***, **, * indicate statistical significance at the 1, 5, and 10% levels, respectively. The numbers in parentheses are standard errors of robust SE

Taking a step further, we examined the gap of health insurance coverage between men and women considering different education levels (see Table 2). The results of the regression show that it was only in the groups of less educated (primary school graduates and below) people that this phenomenon of continuous widening of the gap existed. In groups of senior middle school graduates and below, women’s health insurance coverage rates were all lower than that of men’s but did not show an expanding trend. Among higher educated people, women’s health insurance coverage rates were not necessarily lower than that of men’s, signifying that the gender gap in health insurance was not obvious among the old, higher educated people.

**Table 2:** Results of Different Estimation Methods: Based on Population Census in 2015

Dependent Variable: Covered by the Urban Employee Basic Medical Insurance (UEBMI) or Not				
	(1) Primary School Graduates and below	(2) Junior Middle School Graduates	(3) Senior Middle School Graduates	(4) University Graduates and above
Female	−0.0241 (0.0170)	0.195*** (0.0191)	0.229*** (0.0340)	0.211*** (0.0758)
Female *age [15,25)	−0.0506 (0.0337)	− 0.282*** (0.0239)	− 0.375*** (0.0425)	− 0.219*** (0.0842)
Female *age [25,35)	− 0.0156 (0.0263)	−0.343*** (0.0224)	− 0.548*** (0.0384)	− 0.323*** (0.0792)
Female *age [35,45)	0.0592*** (0.0230)	−0.304*** (0.0218)	− 0.373*** (0.0376)	− 0.183** (0.0814)
Female *age [45,50)	0.0753*** (0.0271)	−0.223*** (0.0267)	− 0.168*** (0.0410)	− 0.169* (0.0992)
Female *age [55,60)	− 0.0753*** (0.0246)	− 0.130*** (0.0325)	0.0693 (0.0612)	− 0.134 (0.123)
Female *age [60,65)	− 0.152*** (0.0262)	− 0.418*** (0.0391)	− 0.202*** (0.0670)	− 0.167 (0.133)
Female *age [65,70)	− 0.176*** (0.0263)	− 0.295*** (0.0515)	− 0.134 (0.0826)	− 0.197 (0.133)
Female *age [70,75)	− 0.182*** (0.0272)	− 0.283*** (0.0708)	−0.186 (0.125)	− 0.255 (0.174)
Female *age [75,80)	−0.285*** (0.0312)	− 0.180 (0.111)	−0.573*** (0.170)	0.395 (0.295)
Female *age [80 +, 100)	−0.380*** (0.0339)	−0.847*** (0.136)	− 0.675*** (0.210)	−0.271 (0.335)
X	YES	YES	YES	YES
County	YES	YES	YES	YES
Observations	771,963	755,007	248,055	128,602

Notes: 1) The control age group is [50, 55)

2) The control variable X and fixed effects of cities have been added to all analyses

3) ***, **, * indicate statistical significance at the 1, 5, and 10% levels, respectively. The numbers in parentheses are standard errors of robust SE

### Gender gap in health insurance coverage: based on survey data of 1% of the population in 2015 (sample B)

In the following analysis, we further used Sample B.

Figure 1 indicates that the results are similar to those generated from using Sample A, except that the turning point here has shifted to the age of 60. Below the age of 60, men and women’s UEBMI coverage rates were almost equivalent, and the coverage rate for young women was even higher than that for men. Meanwhile, the proportion of women aged above 60 covered under UEBMI was much lower than that of men. Considering the level of education (Fig. 3), the coverage rates for men and women below 60 were almost equivalent and not influenced by the level of education. Meanwhile, for those aged over 60, only for the less educated groups (primary school graduates and below) had a significantly lower coverage of women than men, and this gap continued to grow with age.

We applied model (1) on Sample B to perform the same regression and report the corresponding results in Tables 3 and 4. As seen from the tables, for those below the age bracket of [60, 65), there is no clear trend in these interaction term coefficients, but the coefficients continuously increase (with a negative sign) for those above this age bracket. This indicates that the gap in UEBMI coverage rates between women and men has been getting wider and wider for those above the age of 60. Moreover, considering the level of education, the regression analysis shows that this phenomenon of continuous increase in insurance coverage gap for the [60, 65) age group and above only exists among the less educated population (primary school graduates and below).

**Table 3:** Gender Gap in Health Insurance Coverage: Based on Population Census in 2015

Urban Employee Basic Medical Insurance (UEBMI) (Dependent Variable: Covered by UEBMI or Not)			
	(1)	(2)	(3)
Female	−0.177*** (0.0226)	− 0.258*** (0.0241)	0.240*** (0.0270)
Female *age [15,25)	0.526*** (0.0344)	0.558*** (0.0366)	−0.0568 (0.0401)
Female *age [25,35)	0.238*** (0.0269)	0.255*** (0.0289)	−0.232*** (0.0328)
Female *age [35,45)	0.0418 (0.0264)	0.0716** (0.0282)	−0.244*** (0.0319)
Female *age [45,50)	−0.0382 (0.0294)	0.00757 (0.0314)	−0.229*** (0.0350)
Female *age [50,55)	0.0610** (0.0306)	0.104*** (0.0327)	−0.0182 (0.0365)
Female *age [55,60)	0.179*** (0.0335)	0.202*** (0.0358)	0.165*** (0.0405)
Female *age [65,70)	−0.190*** (0.0349)	−0.211*** (0.0372)	− 0.221*** (0.0416)
Female *age [70,75)	−0.371*** (0.0389)	− 0.386*** (0.0412)	−0.386*** (0.0472)
Female *age [75,80)	−0.373*** (0.0436)	−0.411*** (0.0460)	− 0.294*** (0.0543)
Female *age [80 +, 100)	−0.707*** (0.0464)	− 0.693*** (0.0491)	−0.454*** (0.0572)
X	No	YES	YES
County	No	No	YES
Observations	838,544	838,544	838,544

Notes: 1) The control age group is [60, 65)

2) The control variable X includes registered permanent residence, marital status, educational level, nationality, and so on. County represents fixed effects after controlling for counties

3) ***, **, * indicate statistical significance at the 1, 5, and 10% levels, respectively. The numbers in parentheses are standard errors of robust SE

**Table 4:** Gender Gap in Health Insurance Coverage in 2015 by Educational Levels

Dependent Variable: Covered by Urban Employee Basic Medical Insurance (UEBMI) or Not				
	(1) Primary School Graduates and below	(2) Junior Middle School Graduates	(3) Senior Middle School and Technical Secondary School Graduates	(4) University Graduates and above
Female	0.0157 (0.0427)	0.385*** (0.0442)	0.188** (0.0938)	−0.141 (0.180)
Female *age[15,25)	−0.200 (0.341)	−0.435*** (0.0868)	− 0.0729 (0.105)	0.517*** (0.184)
Female *age[25,35)	−0.181 (0.135)	−0.468*** (0.0592)	− 0.246** (0.0992)	0.249 (0.182)
Female *age[35,45)	−0.214*** (0.0730)	−0.422*** (0.0512)	− 0.0973 (0.0990)	0.193 (0.185)
Female *age[45,50)	−0.266*** (0.0766)	−0.377*** (0.0549)	0.0547 (0.106)	0.0980 (0.195)
Female *age[50,55)	−0.158** (0.0804)	−0.136** (0.0569)	0.243** (0.107)	0.194 (0.203)
Female *age[55,60)	0.182** (0.0731)	0.170*** (0.0638)	0.150 (0.116)	0.0617 (0.242)
Female *age[65,70)	−0.327*** (0.0603)	−0.114 (0.0782)	− 0.109 (0.166)	0.0317 (0.296)
Female *age[70,75)	−0.583*** (0.0643)	−0.333*** (0.0970)	− 0.0613 (0.179)	0.135 (0.327)
Female *age[75,80)	−0.533*** (0.0692)	−0.466*** (0.133)	0.0874 (0.225)	0.0465 (0.356)
Female *age[80 +, 100)	−0.734*** (0.0693)	−0.386** (0.173)	− 0.568** (0.251)	−0.515 (0.398)
X	YES	YES	YES	YES
County	YES	YES	YES	YES
Observations	306,558	324,610	126,747	80,629

Notes: 1) The control age group is [60, 65)

Combining the results above, the study using Sample B yielded almost the same results compared with the analysis based on Sample A. The only difference is the age at which the change in the gender gap in health insurance coverage occurred, which increased from age 50 to age 60. This suggests that the situation (i.e., failing to get insurance coverage) has not changed 10 years later for part of the women aged above 50.

2) The control variable X and fixed effects of counties have been added to all analyses.

3) ***, **, * indicate statistical significance at the 1, 5, and 10% levels, respectively. The numbers in parentheses are standard errors of robust SE.

### Gender insurance gap before and after the implementation of the UEBMI reform

Before the UEBMI reform in 1998, labor insurance, in effect, had covered family dependents. Thus, women, even though without a job, were able to obtain medical insurance benefits through the coverage of their working spouse. However, after the reform, the UEBMI’s feature of being tied to work and not including other family members, made it harder than before for these unemployed women to obtain health insurance, which might have led to a widening of the gender gap in health insurance coverage. To verify this conjecture, we used Sample C, covering data before and after the 1998 reform, which enabled us to compare changes in health insurance between men and women.

**Fig. 4:**
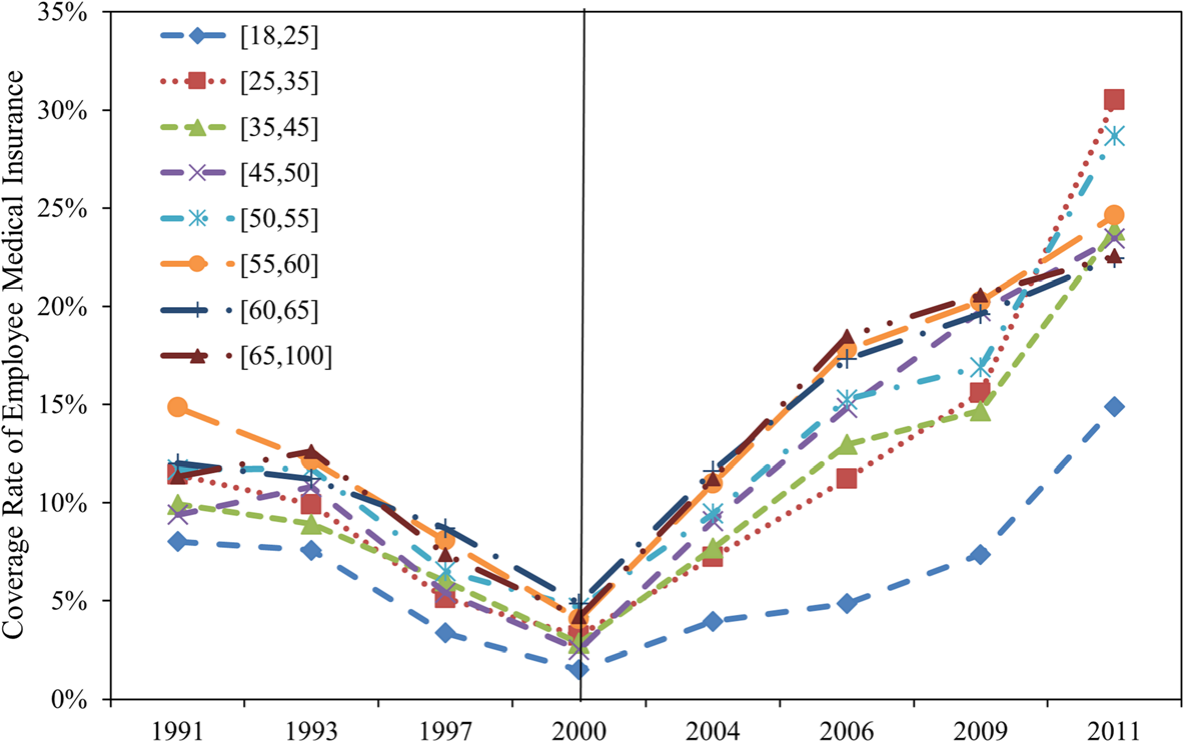
The Urban Employee Basic Medical Insurance (UEBMI) Coverage Rate: 1991–2011

First, the changes in the UEBMI coverage rate from 1991 to 2011 reappear in Fig. 4 (what should be noted here is that we combined labor insurance and various forms of employee medical insurance modes before 1998 into UEBMI for a comparison before and after that year). Figure 4 shows that there was a significant decline in the health insurance coverage rates for all age groups before 2000 but these began to increase rapidly starting from the implementation of the UEBMI. In 2011, the UEBMI had already covered 25% of the population.

**Fig. 5:**
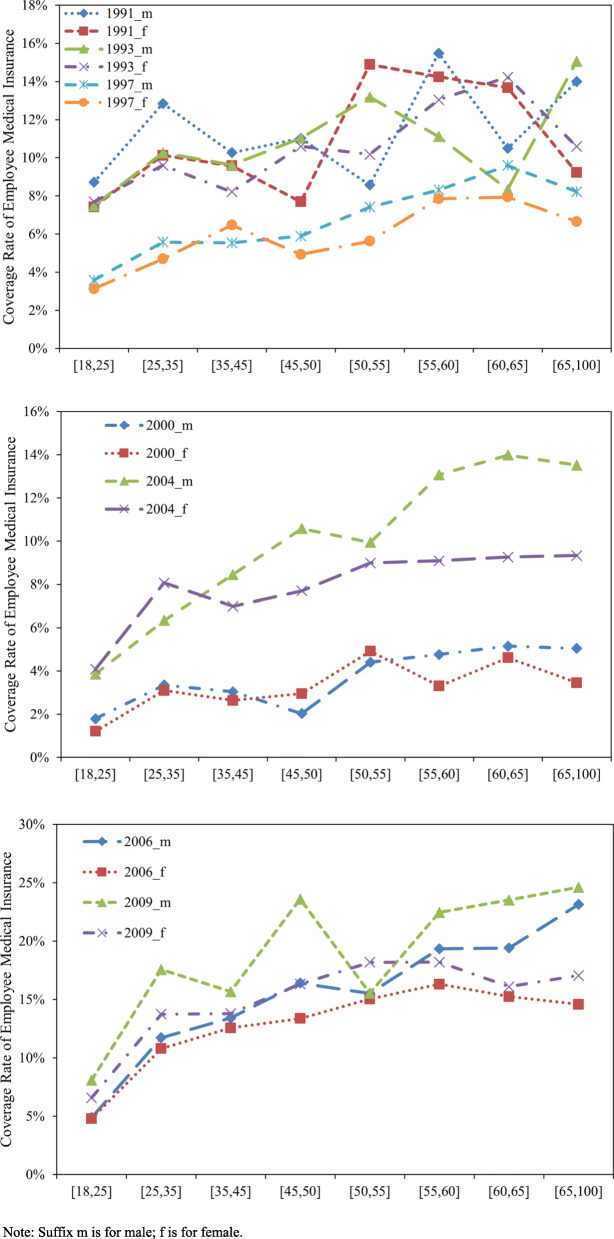
Gender Gap of The Urban Employee Basic Medical Insurance (UEBMI) Coverage Rate: 1991–2009

Meanwhile, Fig. 5 shows no obvious difference in health insurance coverage rates between men and women among the investigated population in 1991 and 1993. However, the increasingly apparent gender gap was observed in the survey samples of 1997 and later, in 2000, 2004, 2006 and 2009, this gap mainly existed among the older population.

## Discussion

### Possible reasons for gender difference in health insurance coverage

The analysis shows that there were significant gender differences in health insurance, especially among the elderly, because of the unique health insurance system in China. The health insurance reform in 1998, which tied health insurance to work, resulted in more unemployed women lacking access to health insurance. According to a study based on the 1998 and 2003 National Health Service Survey data by Xu et.al [11], inequities in access to health insurance has intensified among different populations since 1997 because of the UEBMI reform. Moreover, women are significantly less likely to be insured compared to the pre-reform period. Theoretically, the unemployed or informal employed can also be enrolled in the UEBMI, except that they must bear a higher contribution. According to the regulation, “For the self-employed and freelancers, the health insurance premium, consisting of the work unit’s contribution and that of the individual, should all be paid by the individual.”^1^ Although it is theoretically possible for a person to enroll in insurance, that is, to make a one-off social insurance contribution individually, a considerable amount of health insurance premium must be paid, which might be unaffordable for many families. Moreover, the requirement to pay premiums for at least 15 years reduced the probability of coverage for those over 35 years of age, when the retirement age in China was 50. Enrolment for those aged above 35 meant that premiums would continue to be paid even after retirement, with the costs borne entirely by the individual. This explains why the gender gap in health insurance coverage increased with age and why gender differences were concentrated in the over-50 years old sample in 2005. Why do elderly women face more difficulties in obtaining health insurance? This is mainly a result of differences in labor participation. It is more likely for elderly women to be laid off or lose their jobs, and more difficult for them to be reemployed. For instance, with the reform of the State-Owned Enterprises (SOEs) in the late 1990s, a large number of workers were laid off or lost their jobs when enterprises were facing increasingly competition [11–14].

### Gender differences among different educational groups

Regarding subgroups by education, gender differences in health insurance are mainly concentrated in the low-education group. The empirical study of Appleton et.al [15] indicated that women and people who are less educated, less skilled, and middle-aged are more likely to be laid off. During the critical period of the reform of China’s SOEs, less educated women were not competitive in the labor market, which made them vulnerable to changes in the labor market. During this period, a large number of SOEs were restructured or went bankrupt, which caused a significant number of workers to lose their jobs. The group of women with low education was the most affected. On the one hand, there was greater demand for male labor, as China was primarily developing industry at that time. On the other hand, low-educated women were mainly employed in jobs that were highly substitutable with and highly vulnerable to technological advances [16]. The loss of a job meant the loss of health insurance, which increased the medical burden on women and reduced accessibility of health resources. Furthermore, inequitable access to medical resources might directly affect the health of the population.

### Gender differences in health insurance and health status

In the literature, major disputes have always existed regarding the relationship between health insurance and health status. In a few studies, a significant impact of health insurance spending on people’s health status and mortality has been found [17, 18]. However, most empirical studies have indicated that this effect is small [19–21]. In the context of universal health insurance in China, numerous recent empirical studies have shown that the gradual implementation of basic health insurance has significantly increased access to medical services and improved the health status of the insured [22–24].

However, with the low proportion of older women being covered by the UEBMI, it was difficult for most of them to receive the necessary medical services. This suggests that the gender gap in health insurance exerted influence on people’s health status mainly through its effects on the quality of medical services, including patients’ ability to select better hospitals, medicine, and treatments. Specifically, women’s higher incidence of nonfatal chronic diseases was probably a result of their not having access to medical services that have the same quality as those enjoyed by men.

**Fig. 6:**
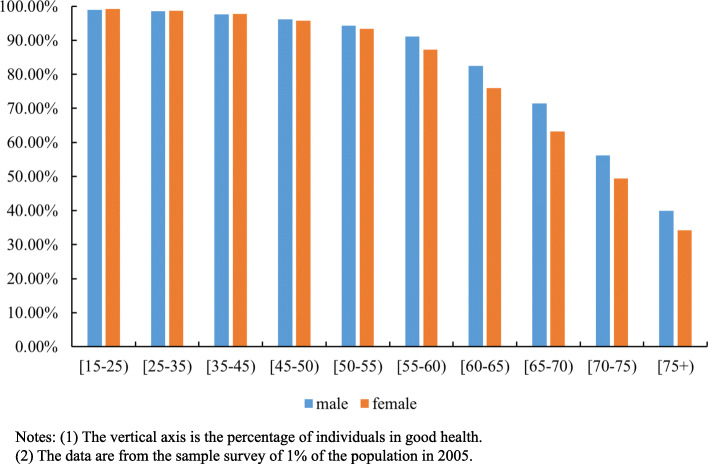
Gender Health Gap

Although women tend to live longer compared with men, many studies have shown that their health condition is worse [25–27]. According to the findings of existing literature, the poorer health status of women in developed countries is a result of their higher possibility of suffering from nonfatal chronic diseases such as arthritis, most respiratory illnesses, hypertension, eyesight problems and depression [26]. A new study by Zhang indicated that about 30% of the gender health gap among Chinese residents could be explained by the difference in the social and economic status between men and women, of which the role of education is most important [27]. However, in our study, the following differences were found from the findings in developed countries. While in the western developed countries, citizens’ gender health gap generally tends to disappear gradually, that of the Chinese citizens’ do not shrink with age (Fig. 6). As indicated in Fig. 6, women of all ages reported worse health than men, but this gap was especially significant among older people (above 60).

### Establish a unified health insurance system

Although China had established a universal health insurance system, there are still obvious inequities remain in different insurance types. The main differences among these major types of insurance relate to their financing and benefits. The UEBMI has the highest level of financing and benefits—significantly higher than those of the other two. Firstly, regarding the conditions for enrollment, the UEBMI is mainly available to people who are formally employed, and those who are unemployed or informally employed are mainly covered by the URBMI and the NCMS. Considering the generally low income of URBMI and NCMS enrollees, the central and local governments subsidize these groups of enrollees. Specifically, both the URBMI and NCMS are financed by individual contributions and subsidies from the central and local governments. The UEBMI is financed through enterprise and employee contributions. The premium rate is 8% of the annual payroll, of which 6% comes from the employer and 2%, from the employee. Secondly, the annual premiums vary widely among insurance types. The annual per capita premium for the UEBMI in 2018 was 5808.3 yuan, while that for the URBMI and NCMS was only 763.4 yuan [28]. Thirdly, in terms of reimbursement rates, the benefit package for the UEBMI includes inpatient and outpatient care, while for the URBMI reimbursements are for inpatient medical expenses only. Meanwhile, the annual reimbursement ceiling is six times of the average wage of employees in the city for the UEBMI and six times the disposable income of residents for the URBMI [3]. What’s more, Regarding the reimbursement ratio, Zhao et al. [29] found that the actual reimbursement ratio for the UEBMI was 75%, while that for the URBMI was 50%. In other words, the portion of the low-income population that is unemployed or informally employed bears more of the burden of health care costs, which clearly results in inequity in the distribution of medical resources due to inequitable health insurance [30–32]. Moreover, some studies have found that the type of the health insurance affects equity in the use of health care services, such that the use of health care services differs among residents covered by different insurance policies [33–36].

The significant gender differences in the coverage of UEBMI in China, especially in the elderly population, may lead this vulnerable group to reduce their consumption of medical resources and further affect their physical health. Low reimbursement rates will reduce the utilization of medical resources by residents, mainly women and the elderly, who are affected by the UEBMI reform. Therefore, in the future improvement of the health insurance system, it is also necessary to improve the benefit level of URBMI and NCMS to reduce the gap between different insurances. Further, it is important to eliminate inequities between different health insurance types. It is generally agreed that China should merge the three schemes into one universal and unified social medical insurance scheme [37–39].

## Conclusion

From the perspective of health insurance reform, this paper explains the gender gap of health insurance and the reason for the wider gap among older people. Our study indicates that since the UEBMI in China mainly covers people with formal jobs, the lower labor participation rate (even much lower in formal jobs) of women leads to their greater difficulty in obtaining health insurance. However, since older women were more likely to be fired or less likely to find a job during the UEBMI reform, they were less likely to be insured. In fact, it was the combined effect of the UEBMI system and the labor market conditions at that time that resulted in a lower proportion of older women obtaining UEBMI.

We used a large volume of data from a demographic survey to verify our conjecture. First, based on the data from the survey of 1% of the population in 2005 and in 2015, it is found that the proportion of women aged over 50 (2005) or 60 (2015) covered under the UEBMI was much lower than that of men, while the gap between young women and men was comparatively narrower. Furthermore, using the 1991–2011 data of CHNS, we find that the disadvantages of women in health insurance coverage only emerged after the implementation of the UEBMI reform in 1998. This further verifies our previous conjecture.

## Supplementary Information

**Additional file 1: Table** **1****A.** Gender Gap in Health Insurance Coverage: Based on Population Census in 2005. **Table** **2****A.** Gender Gap in Health Insurance Coverage: Based on Population Census in 2015.

## Data Availability

The datasets generated and/or analysed during the current study are not publicly available due the data confidentiality agreement, but are available from the corresponding author on reasonable request.

## Abbreviations

SHI: Social health insurance
UEBMI: Urban Employee Basic Medical Insurance
URBMI: Urban Resident Basic Medical Insurance
NCMS: New Cooperative Medical Scheme
CHNS: China Health and Nutrition Survey
SOEs: State-owned Enterprises

## Footnotes

1 Participants who are originally unemployed (including individuals and freelancers) and need to pay basic medical insurance premium for an accumulative total of 15 years stop the payment when they reach the statutory retirement age. If the payment of basic medical insurance premium is for less than the accumulative total of 15 years at the statutory retirement age, participants should continue the payment as required until 15 years thereafter. For reasons such as bankruptcy, restructuring, and institutional reform of state-owned or collective units (including enterprises, the Party, government organizations, and institutions), employees who have left and become self-employed personnel or freelancers should pay the premium continuously (as required before completing formalities of retirement) until the retirement age.
