# Supplementary material for: Gender differences in health insurance coverage in China
Source: Int J Equity Health. 2021 Feb 1;20:52. doi: 10.1186/s12939-021-01383-9 (PMC7852118; doi:10.1186/s12939-021-01383-9)
Supplement: Supplementary file 1 — Additional file 1: Table 1A. Gender Gap in Health Insurance Coverage: Based on Population Census in 2005. Table 2A. Gender Gap in Health Insurance Coverage: Based on Population Census in 2015. [file 12939_2021_1383_MOESM1_ESM.doc]

**Appendix**

**Table 1A: Gender Gap in Health Insurance Coverage: Based on Population Census in 2005**

|  | Urban Employee Basic Medical Insurance (UEBMI)  (Dependent Variable: Covered by UEBMI or Not) | | | |  |
| --- | --- | --- | --- | --- | --- |
|  | | OLS | logistic | | |
| Female | | 0.00859***  (0.00194) | | 0.0548***  (0.0110) | |
| Female *age  [15,25) | | -0.0220***  (0.00254) | | -0.148***  (0.0161) | |
| Female *age  [25,35) | | -0.0309***  (0.00232) | | -0.199***  (0.0138) | |
| Female *age  [35,45) | | -0.0187***  (0.00227) | | -0.119***  (0.0132) | |
| Female *age  [45,50) | | -0.00262  (0.00276) | | -0.0191  (0.0158) | |
| Female *age  [55,60） | | -0.0203***  (0.00295) | | -0.121***  (0.0169) | |
| Female *age  [60,65） | | -0.0427***  (0.00324) | | -0.250***  (0.0186) | |
| Female *age  [65,70） | | -0.0439***  (0.00344) | | -0.249***  (0.0197) | |
| Female *age  [70,75） | | -0.0506***  (0.00372) | | -0.277***  (0.0211) | |
| Female *age  [75,80） | | -0.0624***  (0.00442) | | -0.344***  (0.0253) | |
| Female *age  [80+，100） | | -0.0754***  (0.00490) | | -0.426***  (0.0284) | |
| X | | YES | | YES | |
| City | | YES | | YES | |
| Observations | | 1,903,627 | | 1,903,627 | |

Notes: 1) The control age group is [50, 55).

2) The control variable X includes registered permanent residence, marital status, educational level, nationality, and so on. City represents fixed effects after controlling for cities.

3) ***, **, * indicate statistical significance at the 1%, 5%, and 10% levels, respectively. The numbers in parentheses are standard errors of robust SE.

**Table 2A: Gender Gap in Health Insurance Coverage: Based on Population Census in 2015**

|  | Urban Employee Basic Medical Insurance (UEBMI)  (Dependent Variable: Covered by UEBMI or Not) | | | |  |
| --- | --- | --- | --- | --- | --- |
|  | | OLS | logistic | | |
| Female | | 0.0151***  (0.0024) | | 0.240***  (0.0270) | |
| Female *age  [15,25) | | -0.0207***  (0.0028) | | -0.0568  (0.0401) | |
| Female *age  [25,35) | | -0.0094***  (0.0029) | | -0.232***  (0.0328) | |
| Female *age  [35,45) | | -0.0129***  (0.0028) | | -0.244***  (0.0319) | |
| Female *age  [45,50) | | -0.0109***  (0.0030) | | -0.229***  (0.0350) | |
| Female *age  [55,60） | | 0.00580*  (0.0033) | | -0.0182  (0.0365) | |
| Female *age  [60,65） | | 0.0204***  (0.0038) | | 0.165***  (0.0405) | |
| Female *age  [65,70） | | -0.0251***  (0.0038) | | -0.221***  (0.0416) | |
| Female *age  [70,75） | | -0.0428***  (0.0042) | | -0.386***  (0.0472) | |
| Female *age  [75,80） | | -0.0442***  (0.005) | | -0.294***  (0.0543) | |
| Female *age  [80+，100） | | -0.0707***  (0.0051) | | -0.454***  (0.0572) | |
| X | | YES | | YES | |
| City | | YES | | YES | |
| Observations | | 838,544 | | 838,544 | |

Notes: 1) The control age group is [50, 55).

2) The control variable X includes registered permanent residence, marital status, educational level, nationality, and so on. City represents fixed effects after controlling for cities.

3) ***, **, * indicate statistical significance at the 1%, 5%, and 10% levels, respectively. The numbers in parentheses are standard errors of robust SE.
